# *Liberibacter crescens* biofilm formation in vitro: establishment of a model system for pathogenic ‘*Candidatus* Liberibacter spp.’

**DOI:** 10.1038/s41598-019-41495-5

**Published:** 2019-03-26

**Authors:** Eber Naranjo, Marcus V. Merfa, Virginia Ferreira, Mukesh Jain, Michael J. Davis, Ofir Bahar, Dean W. Gabriel, Leonardo De La Fuente

**Affiliations:** 1 Department of Entomology and Plant Pathology, Auburn University, Auburn, USA; 2 Bioscience Department, College of Chemistry, University of the Republic, Montevideo, Uruguay; 3 Department of Plant Pathology, University of Florida, Gainesville, USA; 4 Citrus Research and Education Center, University of Florida, Gainesville, USA; 5 Department of Plant Pathology and Weed Research, ARO - Volcani Center, Bet-Dagan, Israel

## Abstract

The *Liberibacter* genus comprises insect endosymbiont bacterial species that cause destructive plant diseases, including Huanglongbing in citrus and zebra chip in potato. To date, pathogenic ‘*Candidatus* Liberibacter spp.’ (CLs) remain uncultured, therefore the plant-associated *Liberibacter crescens* (Lcr), only cultured species of the genus, has been used as a biological model for *in vitro* studies. Biofilm formation by CLs has been observed on the outer midgut surface of insect vectors, but not in planta. However, the role of biofilm formation in the life cycle of these pathogens remains unclear. Here, a model system for studying CLs biofilms was developed using Lcr. By culture media modifications, bovine serum albumin (BSA) was identified as blocking initial cell-surface adhesion. Removal of BSA allowed for the first time observation of Lcr biofilms. After media optimization for biofilm formation, we demonstrated that Lcr attaches to surfaces, and form cell aggregates embedded in a polysaccharide matrix both in batch cultures and under flow conditions in microfluidic chambers. Biofilm structures may represent excellent adaptive advantages for CLs during insect vector colonization helping with host retention, immune system evasion, and transmission. Future studies using the Lcr model established here will help in the understanding of the biology of CLs.

## Introduction

*‘Candidatus* Liberibacter’ species are the causal agents of devastating plant diseases worldwide that include Huanglongbing (HLB) or citrus greening, and zebra chip (ZC) of potato^1,2^. HLB was first described in Guangdong Province (China) in the late 19th century and is currently a serious threat to major citrus-producing areas worldwide^1,3^. In the US, HLB was first detected in 2005 in Florida and since then it has seriously impacted the US citrus industry with ~$300 million in losses per year^4^.

HLB is associated with three different species that include the α-proteobacteria ‘*Ca*. Liberibacter asiaticus’ (CLas), ‘*Ca*. L. americanus’ (CLam), and ‘*Ca*. L. africanus’ (CLaf)^1^. CLas and CLam are circulative endosymbionts in their insect vector, the Asian citrus psyllid (ACP) *Diaphorina citri*, and CLaf is an endosymbiont of the African citrus psyllid *Trioza erytreae*^2^. ZC is a newly-emerged disease associated with ‘*Ca*. L. solanacearum’ (CLso)^5,6^. Since its first detection in tomato and potato, CLso has been reported to cause vegetative disorders in pepper^7^, tobacco^8^, carrot^8^, celery^9^, bittersweet^10^, silverleaf nightshade (*Solanum elaeagnifolium*)^11^, and more recently in chervil, fennel, parsley and parsnip^12^. In 2009, ZC was prevalent in most potato-producing states in the US, and reached epidemic levels in 2011 in the Pacific Northwest states^13^.

The pathogenic species of ‘*Ca*. Liberibacter’ share specific features that make them difficult to study: first, all are well adapted insect endosymbionts that readily traverse insect membranes^14^; second, all of them are exclusively phloem-limited and intracellular within their plant hosts^2^; and third, they have suffered extensive reductions in genome content to ca. 1.2 Mb^15^, making them highly dependent on their hosts. To date none of the pathogenic ‘*Ca*. Liberibacter’ species have been cultured *in vitro*^1^.

*Liberibacter crescens* (Lcr) strain BT-1 is the only cultured wild-type strain of the *Liberibacter* genus, with a genome size of 1.4 Mb, larger than those of ‘*Ca*. Liberibacter spp’^16^. BT-1 was isolated from the phloem sap of a defoliating mountain papaya in Puerto Rico in 1995^17^. Despite numerous efforts, Lcr is not known to be pathogenic^17^, and attempts to inoculate this strain into several plants have been unsuccessful (Michael J. Davis, unpublished results). However, the ability to culture Lcr *in vitro* allows development of Lcr strains as biological models to study and predict the pathogenic but uncultured ‘*Ca*. Liberibacter spp.’. Lcr has proven to be genetically tractable for both knockout and knock-in mutations and therefore useful for functional genomic studies of ‘*Ca*. Liberibacter spp’^18–20^, thereby leveraging the results of bioinformatic studies^1,6,15^.

Biofilms are assemblages of microorganisms attached to a solid surface and encased in an extracellular polymeric matrix^21,22^. These surface-associated bacterial biofilm communities are widespread in all types of natural environments, and are often more abundant than planktonic bacteria in pathogenic ecosystems^21^. Biofilm formation has not been observed in association with ‘*Ca*. Liberibacter spp.’ inside their plant hosts. However, in insect hosts biofilm formation has been observed, but limited to the outer surfaces of the midgut. Fluorescence *in-situ* hybridization (FISH) studies targeting CLas and CLso cells within infected psyllids have shown these pathogens extensively and circulatively colonizing their insect hosts in multiple tissues, penetrating multiple membranes and multiplying both intra- and intercellularly, ultimately forming biofilms extracellularly on the outer, hemolymph side of the insect midgut^23,24^.

In this study we established an *in vitro* model system that demonstrated the capacity of Lcr to attach to surfaces, form cell-cell aggregates and form an extracellular polysaccharide matrix in batch cultures and under flow conditions, all main features of biofilm-forming bacteria. The study of biofilm formation in Lcr as a model for pathogenic ‘*Ca*. Liberibacter spp.’, provides an opportunity to better understand the biological interactions of these bacteria with their hosts, and the factors that govern their movement from intercellular to extracellular environments. This will also inform the design of novel control strategies taking into consideration the protection of biofilms against antibacterial compounds.

## Results

### Cultural factors influence Lcr planktonic and biofilm growth

**Figure 1:**
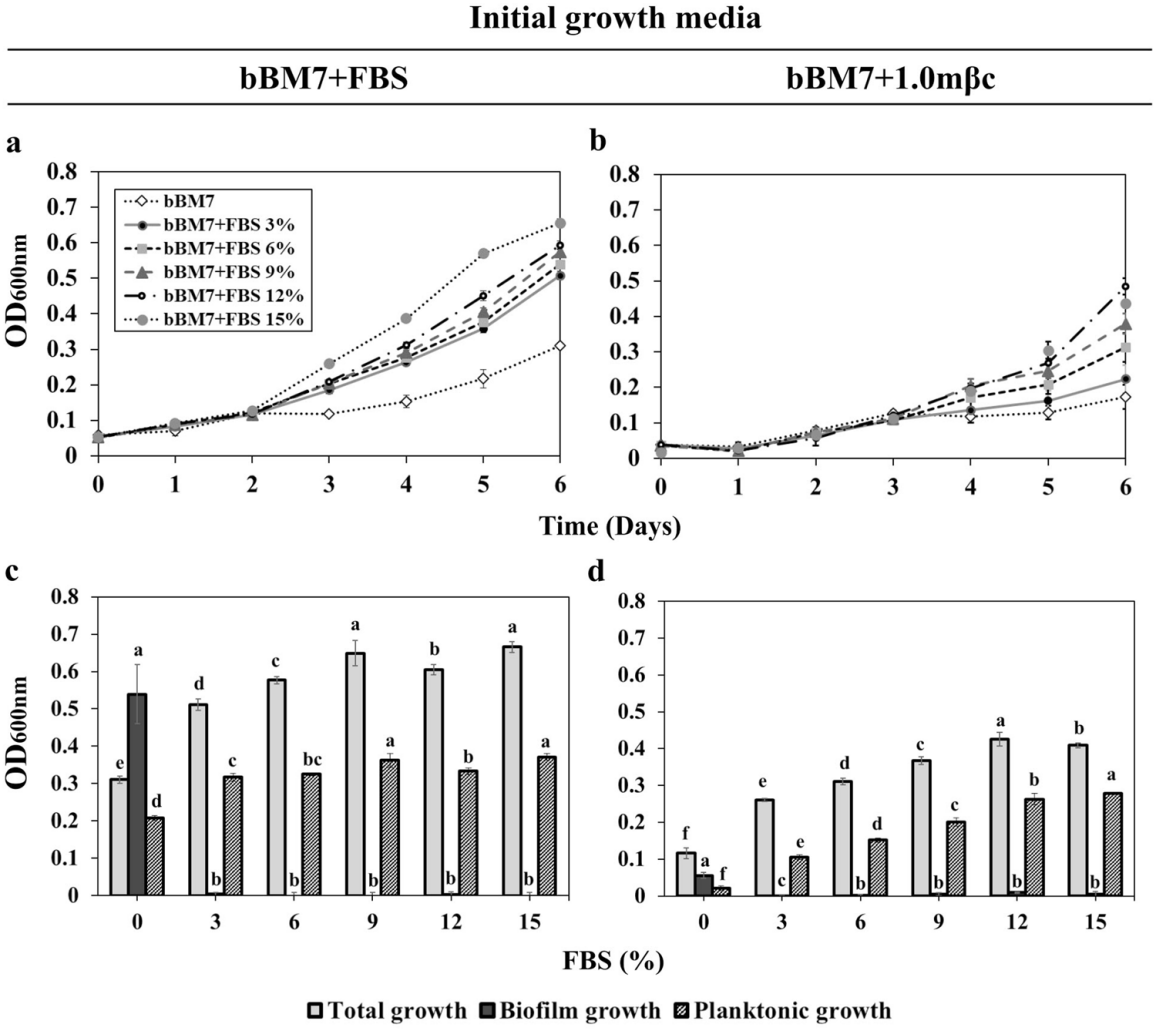
*Liberibacter crescens* (Lcr) growth and biofilm formation under Fetal Bovine Serum (FBS) concentration gradients. A basal medium (bBM7, see Table 2) consisting of BM7 without FBS, was supplemented with FBS to reach final concentrations ranging from 3 to 15%. (**a,b**) Lcr growth curves (n = 6) were started with cells grown either on bBM7 + FBS (**a**) or bBM7 + 1.0 mβc (**b**) agar plates. (**c**,**d**) Lcr total, planktonic and biofilm growth (n = 6) on each FBS concentration was measured at 6 dpi and started from bBM7 + FBS (**c**) or bBM7 + 1.0 mβc (**d**) agar plates. Values on each graph represent mean and standard deviations. In (**c,d**) different letters correspond to statistical significant differences between bars or points of the same pattern according to Fisher’s LSD test (*P* < 0.05). Experiments were repeated two times, and one representative experiment is shown here.

**Figure 2:**
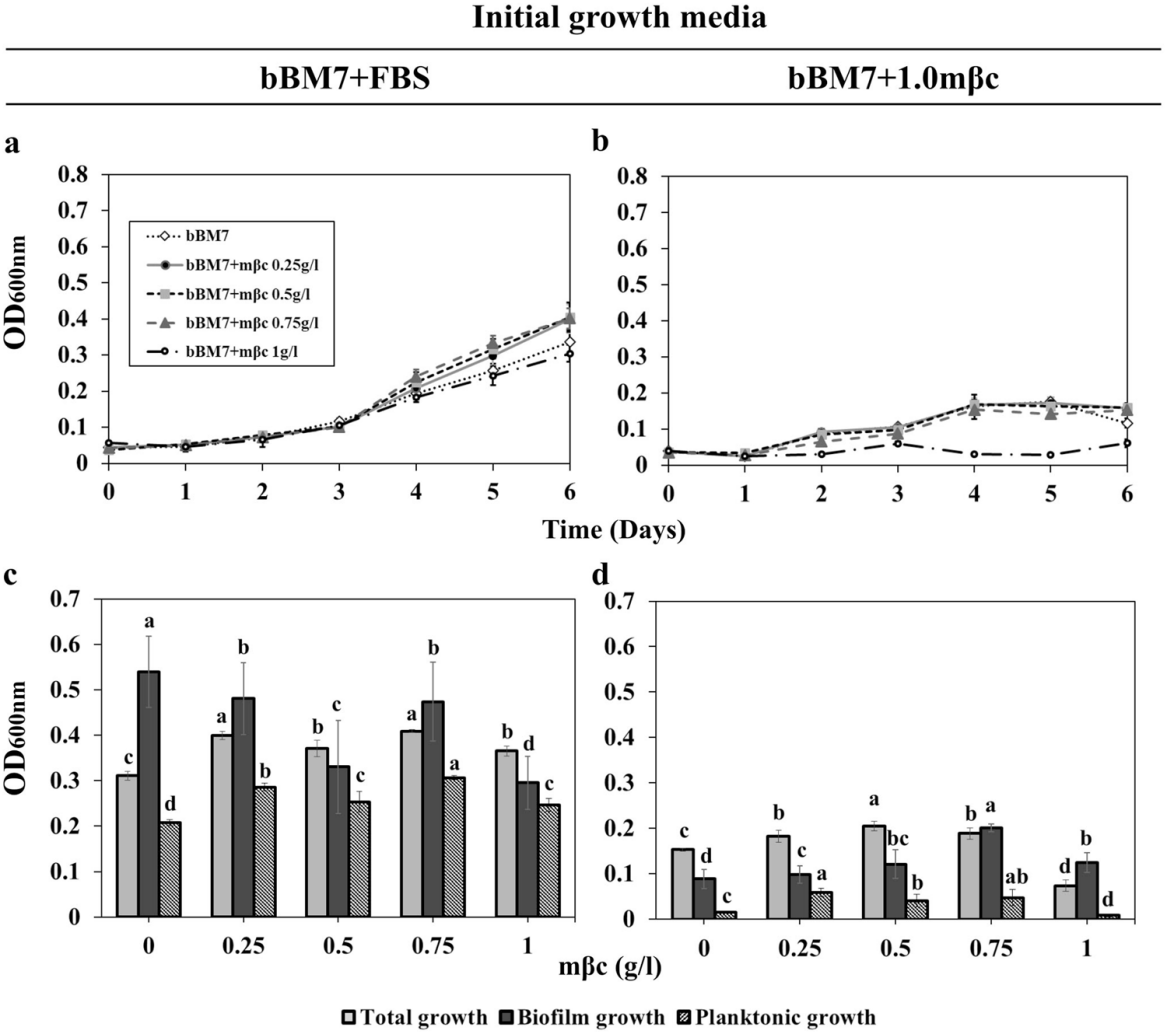
*Liberibacter crescens* (Lcr) growth and biofilm formation under methyl-β-cyclodextrine (mβc) concentration gradients. A basal medium (bBM7, see Table 2) consisting of BM7 without FBS, was supplemented with mβc to reach final concentrations ranging from 0.25 to 1 g/l. (**a,b**) Lcr growth curves (n = 6) were started with cells grown either on bBM7 + FBS (A) or bBM7 + 1.0 mβc (**b**) agar plates. (**c,d**) Lcr total, planktonic and biofilm growth (n = 5–6) on each mβc concentration was measured at 6 dpi and started from bBM7 + FBS (**c**) or bBM7 + 1.0 mβc (**d**) agar plates. Values on each graph represent mean and standard deviations. In (**c,d**), different letters correspond to statistical significant differences between bars or points of the same pattern according to Fisher’s LSD test (*P* < 0.05). Experiments were repeated two times, and one representative experiment is shown here.

To assess the influence of media composition in Lcr growth and biofilm formation, fetal bovine serum (FBS) was removed from the commonly used BM7 medium^16^, and the resulting “basal medium” (bBM7) was supplemented with various concentrations of FBS and methyl-β-cyclodextrin (mβc). To facilitate comparison with the media nomenclature used throughout this manuscript (Table 1), BM7 medium is also referred here as “bBM7 + FBS” that corresponds to bBM7 containing FBS. The effect of the initial media used for the assays was also assessed for each experimental condition, where the regular bBM7 + FBS (a.k.a. BM7) and bBM7 supplemented with 1 g/l of mβc (bBM7 + 1.0 mβc) agar plates (see Table 1 for media formulation notation and composition) were used as precultures. For all experiments that were started from cultures on bBM7 + FBS, a higher growth rate of Lcr was observed than in those that started from cultures on bBM7 + 1.0 mβc agar plates (Figs 1 and 2). FBS concentration positively correlated with Lcr total and planktonic growth (r = 0.82–0.99; *P* < 0.05), regardless of the initial growth media used (Table 2). The optimum FBS concentration for Lcr total growth was 9% for cultures started from bBM7 + FBS (Fig. 1c), and 12% for cultures started from bBM7 + 1.0 mβc (Fig. 1d). Similarly, the highest planktonic growth values were obtained at 9% of FBS for cultures started from bBM7 + FBS (Fig. 1c), while 15% was the optimum FBS concentration for the planktonic growth for cultures started from bBM7 + 1.0 mβc (Fig. 1d). The presence of FBS drastically suppressed Lcr biofilm growth at all concentrations tested, and no significant differences were observed among different FBS concentrations (Fig. 1c,d). Optimal values for total and planktonic growth varied when an mβc gradient was used (Fig. 2c,d). mβc slightly increased total and planktonic growth in experiments starting either from bBM7 + FBS or bBM7 + 1.0 mβc, with optimal concentrations between 0.25 and 0.75 g/l (Fig. 2c,d). Optimal biofilm formation was observed when cultures were pre-grown in bBM7 + FBS agar plates and in the absence of mβc (bBM7) (Fig. 2c). When cultures were started from bBM7 + 1.0 mβc (Fig. 2d) optimal biofilm formation was observed at 0.75 g/l mβc. When cultures were started from bBM7 + FBS no correlation was observed between biofilm formation and mβc concentration (r = −0.50, *P* = 0.89) (Table 2). When bBM7 + 1.0 mβc was used for pre-culture, Lcr biofilm growth increased as mβc concentration increased from 0 to 0.75 g/l (r = 0.91) but with a non-significant correlation (*P* = 0.09) (Table 2).

**Table 1:** Media formulations used in this work.

Reagent^a^	bBM7^b^	bBM7 + FBS (BM7^c^)	bBM7 + 0.75 mβc/bBM7 + 1.0 mβc^d^	bBM7 + BSA^e^
Molecular grade water	700 ml	550 ml	700 ml	700 ml
α-ketoglutaric acid	2 g	2 g	2 g	2 g
ACES buffer	10 g	10 g	10 g	10 g
Potassium hydroxide	3.75 g	3.75 g	3.75 g	3.75 g
TNM-FH insect medium	300 ml	300 ml	300 ml	300 ml
Fetal bovine serum (FBS)		150 ml		
Methyl-β-cyclodextrin (mβc)			0.75/1.0 g^d^	
Bovine serum albumin (BSA)		~3.5 g^f^		3.5 g

^a^All quantities are for 1 L of the corresponding media. For solid media, agar (Difco^TM^. Bectson Dickinson and Company. Sparks, MD. USA) was added at 15 g/l whenever needed.

^b^bBM7: basal BM7 was considered optimal for biofilm formation but displayed the lowest total growth and cell viability values (see results). This study.

^c^BM7 media as described by Leonard *et al*., 2012^29^, also referred here as bBM7 + FBS.

^d^bBM7 + 0.75 mβc was considered optimal for total growth and cell viability (see results). A preliminary version containing 1.0 g/l of mβc (bBM7 + 1.0 mβc) was used for pre-culture of Lcr, see materials and methods for details. This study.

^e^bBM7 supplemented with BSA. This study.

^f^Approximate BSA amount already included in FBS fraction of bBM7 + FBS medium (BM7) according to manufacturer’s information^94^.

**Table 2:** Pearson r coefficent values for the linear regression analysis between total, planktonic,biofilm growth and viability (PRAB) values and different media formulations.

Reagent^a^	Initial growth media	Variables^b^	Pearson r coefficcient	P value
**FBS**	bBM7 + FBS	Total growth/[FBS]	0.87	0.03
		Planktonic growth/[FBS]	0.82	0.04
		Biofilm growth/[FBS]	−0.66	0.16
	bBM7 + 1.0 mβc	Total growth/[FBS]	0.94	<0.001
		Planktonic growth/[FBS]	0.99	<0.001
		Biofilm growth/[FBS]	−0.54	0.26
**mβc**	bBM7 + FBS	Total growth/[mβc]	0.77	0.22
		Planktonic growth/[mβc]	0.79	0.45
		Biofilm growth/[mβc]	−0.50	0.89
		PRAB^c^/[mβc]	0.95	0.06
		PRAB/Total growth	0.68	0.08
		PRAB/Planktonic growth	0.65	0.25
		PRAB/Biofilm growth	−0.74	0.09
	bBM7 + 1.0 mβc	Total growth/[mβc]	0.78	0.22
		Planktonic growth/[mβc]	0.55	0.45
		Biofilm growth/[mβc]	0.91	0.09
		PRAB/[mβc]	0.94	0.06
		PRAB/Total growth	0.92	0.08
		PRAB/Planktonic growth	0.75	0.25
		PRAB/Biofilm growth	0.73	0.27

^a^Media was supplemented with a gradient of concentrations of fetal bovine serum (FBS) or methyl-β-cyclodextrin (mβc). FBS concentrations considered here ranged from 3 to 15% (Fig. 1), while mβc concentrations ranged from 0 to 0.75 g/l.

^b^Total, planktonic and biofilm culture fractions were quantified at 6 dpi.

^c^PRAB: percentage reduction of alamarBlue^®^, was used as a measurement of cell viability.

### Cultural factors influence Lcr viability

**Figure 3:**
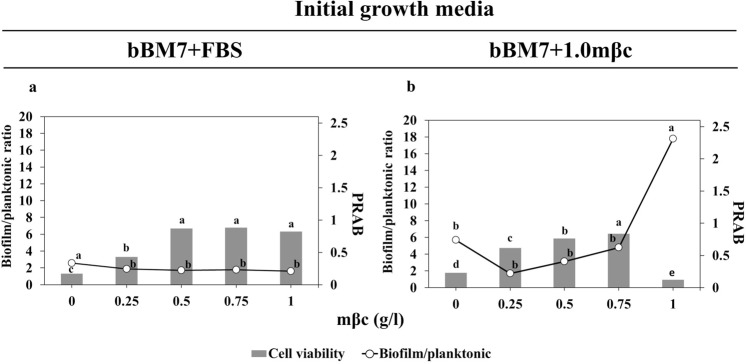
Methyl-β-cyclodextrin (mβc) influence in Lcr biofilm growth and cell viability. (**a**) Cultures started from bBM7 + FBS. (**b**) Cultures started from bBM7 + 1.0 mβc. Left y axes (lines): Biofilm/planktonic ratio values were obtained by averaging values resulting from dividing biofilm growth by planktonic growth for each replicate. Right y axes (bars): percentage reduction of alamarBlue® (PRAB) values as a measurement of cell viability. Different letters on bars (n = 4) and on-line points (n = 6) shown in (**a**), correspond to statistical significant differences according to Fisher’s LSD test (*P* < 0.05). Different letters on bars (n = 4) and on-line points (n = 5–6) shown in (**b**), correspond to statistical significant differences according to Fisher’s LSD test (*P* < 0.05). Experiments were repeated two times, and one representative experiment is shown here.

To quantify the effect of media composition on cell viability, percentage reduction of alamarBlue^®^ (PRAB) was used. Cell viability increased as mβc concentration increased from 0 to 0.75 g/l, regardless of the media formulation in which Lcr was previously grown (r = 0.94–0.95) with a correlation significance level slightly below the 95% confidence interval (*P* = 0.06) (Table 2). No significant differences in cell viability were observed for PRAB values above 0.25 g/l for cultures started from bBM7 + FBS (Fig. 3a). For cultures started from bBM7 + 1.0 mβc, significant differences in cell viability were observed between all mβc concentrations tested, with optimum viability at 0.75 g/l, and a significant decrease at 1 g/l (Fig. 3b). Biofilm/planktonic ratios were significantly higher at the lowest viability values, regardless of the initial growth media (bBM7 + FBS or bBM7 + 1.0 mβc) used (Fig. 3).

The use of bBM7 + FBS agar plates as the pre-culture medium and subsequent growth in liquid bBM7 were the optimum conditions for Lcr biofilm formation, but it was accompanied with low total and planktonic growth and cell viability values (Figs 2c and 3a), which limited the ability to conduct subsequent experiments. Since the combination of bBM7 + FBS for pre-culture and bBM7 + 0.75 mβc resulted in the highest average total and planktonic growth and cell viability (Figs 2c and 3a), and the second highest value for biofilm formation, pre-culturing in bBM7 + FBS followed by culturing in bBM7 + 0.75 mβc were chosen as the optimum protocol for all subsequent assays.

### Cultural factors affect Lcr intracellular ROS generation

**Figure 4:**
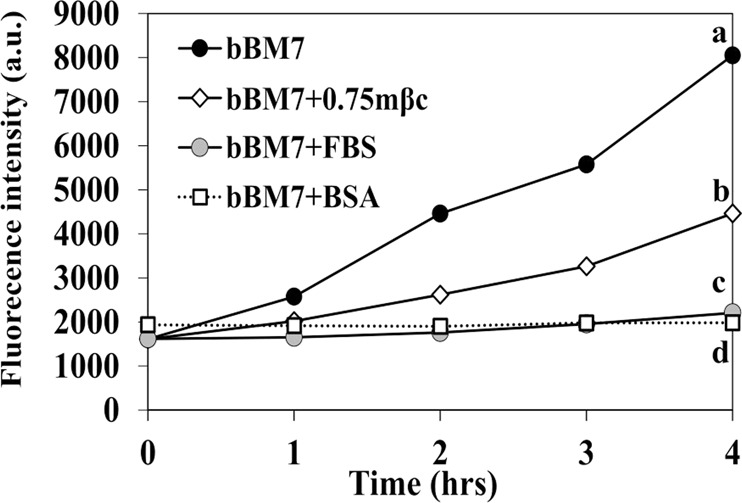
*L. crescens* (Lcr) intracellular ROS production in different media. formulations. Different letters represent significant differences (n = 3) in fluorescence emission of CM-H_2_DCFDA (see methods) at 4 hpi between treatments according to Fisher’s LSD test (*P* < 0.05). Experiments were repeated two times, and one representative experiment is shown here. a.u. = arbitrary units.

Media formulations had a significant effect on Lcr intracellular ROS generation (Fig. 4). A linear increase in intracellular ROS was observed between 0–4 hpi when Lcr was cultured in bBM7 medium. Supplementation with 0.75 g/l mβc mitigated intracellular oxidative stress. Under our assay conditions, both FBS and bovine serum albumin (BSA) maintained constant cellular redox conditions over the time frame analyzed. After one hour of incubation, Lcr intracellular ROS generation was higher in bBM7 than in bBM7 + 0.75 mβc, while bBM7 + FBS and bBM7 + BSA remained around 2000 arbitrary units (a.u.) of fluorescence emission (Fig. 4a). This trend was maintained for the subsequent time points of the experiment (Fig. 4a). After four hours of incubation, Lcr registered the higher ROS generation in bBM7, followed by bBM7 + 0.75 mβc, bBM7 + FBS and bBM7 + BSA, in a decreasing order in fluorescence emission, with significant differences between all the treatments.

### Cultural factors affect Lcr cell-surface attachment and cell-cell aggregation

**Figure 5:**
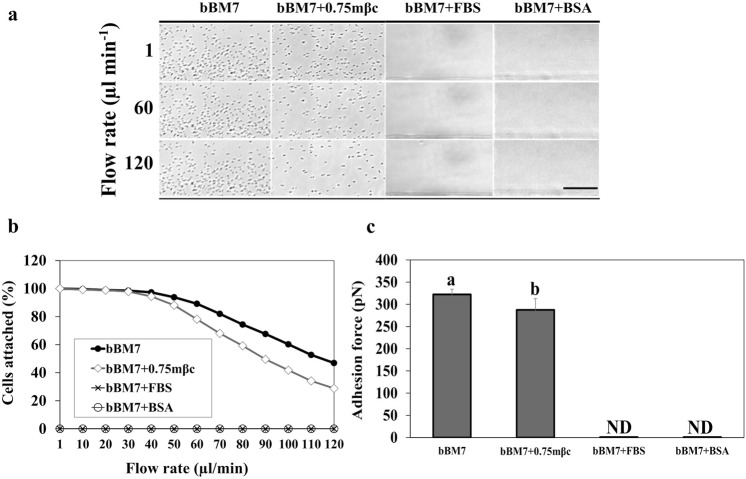
*L. crescens* (Lcr) attachment force assessment in microfluidic chambers (MC) using different media. (**a**) Image sequences showing detachment of Lcr from the MC surface over different flow rates (left) in different media. Scale bar: 20 µm. (**b**) Average percentage of Lcr cells attached to the microfluidic chamber MC surface (n = 6) as a function of the flow rate for each culture treatment. (**c**) Adhesion force for each media formulation. Different letters on bars corresponding to media allowing attachment, indicate statistical significant differences according to Student’s *t*-test *(P* < 0.05*)*. Error bars: standard deviations. N.D. = Non-detected.

Adhesion force measurements showed that Lcr exhibited strongest surface attachment to the microfluidic chambers (MC) in bBM7 medium in the absence of both FBS and mβc (Fig. 5). No attached Lcr cells were observed with the lowest flow rate when inoculated in bBM7 + FBS or bBM7 + BSA suggesting that BSA blocks Lcr cell-surface attachment. Average adhesion force values were 287.45 ± 25 pN for bBM7 + 0.75 mβc and 322.25 ± 12 pN for bBM7 with statistically significant difference (*P* = 0.01) between these treatments (Fig. 5b,c).

**Figure 6:**
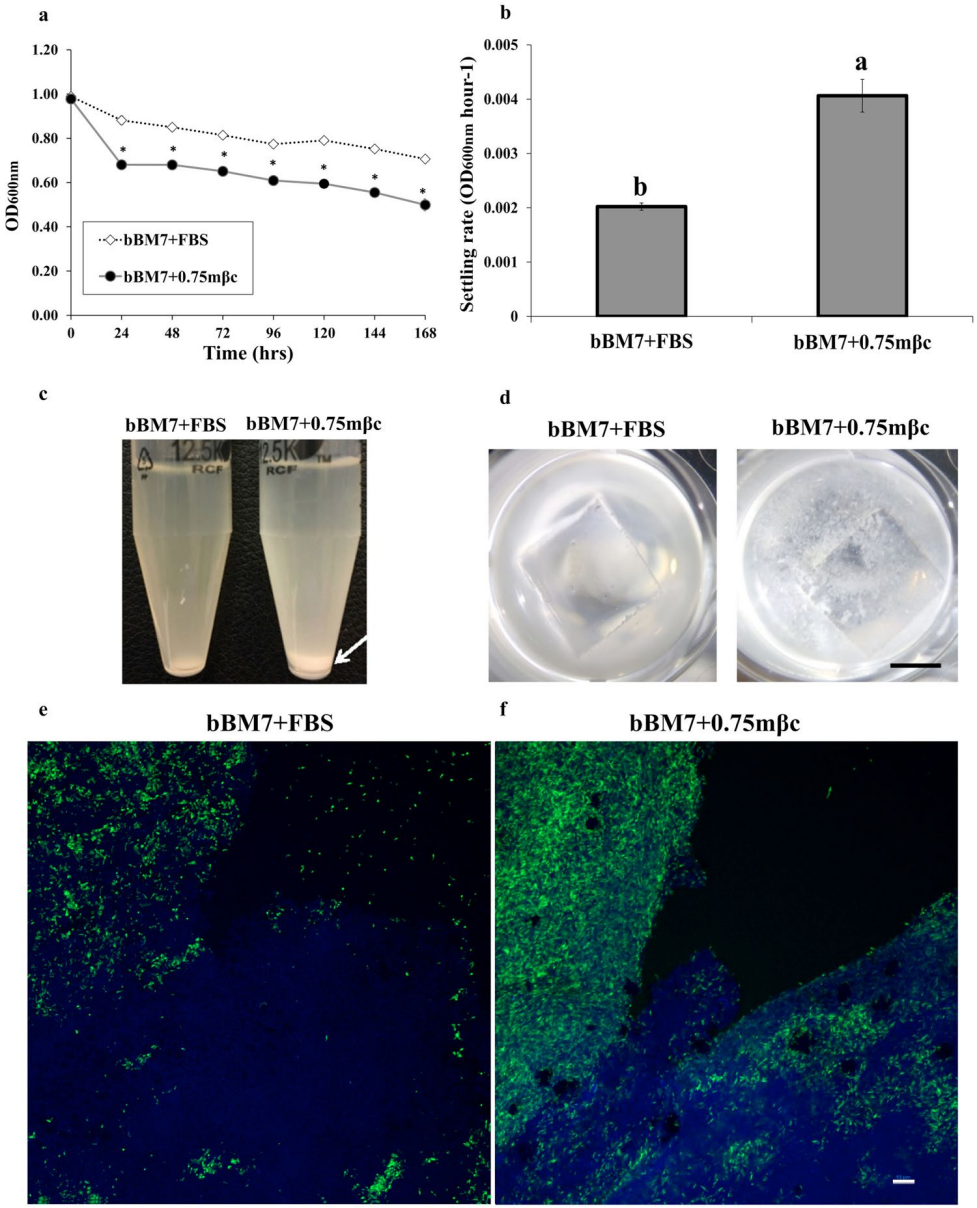
Cell-to-cell aggregation assessment. (**a**) OD_600nm_ as a function of time for *L. crescens* (Lcr) grown in bBM7 + FBS and bBM7 + 0.75 mβc (n = 4). *Represent significant differences between OD_600nm_ values for each treatment at *P* < *0.05* calculated by Student’s *t*-test. (**b**) Settling rate for each growth condition. Different letters on bars correspond to statistical significant differences according to Fisher’s LSD test (*P* < 0.05). Error bars: standard deviations. (**c**) Lcr cells sedimentation at the bottom of the tubes in bBM7 + FBS and bBM7 + 0.75 mβc at 7 dpi. Arrow shows Lcr sediment formation in bBM7 + 0.75 mβc. (**d**) Macroscopic appearance of Lcr cultures in bBM7 + FBS and bBM7 + 0.75 mβc at 7 dpi. Scale bar: 0.4 cm. (**e, f**) Microscopic observation of GFP-Lcr cell aggregates in bBM7 + FBS and bBM7 + 0.75 mβc cultures by CLSM at 7 dpi. Lcr cells were transformed with GFP and are emitting fluorescence in green, polysaccharide matrix is stained in blue by calcofluor white. Scale bar: 10 µm.

Lcr cell-cell aggregation was significantly higher for Lcr cells grown in bBM7 + 0.75 mβc than for cells grown in bBM7 + FBS broth in all time points after 24 h of evaluation (Fig. 6a). The settling rate of Lcr cells grown in bBM7 + 0.75 mβc broth was approximately two-fold higher than cells in bBM7 + FBS broth (Fig. 6b) and was visible as a larger cell sediment in bBM7 + 0.75 mβc than in bBM7 + FBS at 7 dpi (Fig. 6c). Lcr cell-cell aggregates appeared qualitatively different on each media formulation. After a week of incubation, Lcr formed cloud-like cell aggregates in the bottom of wells of all the bBM7 + 0.75 mβc treatments, which were visible with the naked eye, while a fine layer of settled cells was observed in bBM7 + FBS (Fig. 6d). Calcofluor white staining of these cell aggregates from both bBM7 + FBS and bBM7 + 0.75 mβc cultures indicated the presence of an extracellular polysaccharide matrix in both media formulations. Lcr formed bigger and more compact cell aggregates in bBM7 + 0.75 mβc (Fig. 6d,e). In bBM7 + FBS, the extracellular polysaccharide matrix stained by calcofluor white seemed more diffuse and the cells more easily dislodged from the cell aggregates when covered with a coverslip. By contrast, cell aggregates collected from bBM7 + 0.75 mβc medium showed a more intact structure after placing them in a glass sandwich, with the extracellular polysaccharide matrix and cells forming a gel-like compact structure (Fig. 6e).

### Characterization of Lcr biofilm in batch cultures

**Figure 7:**
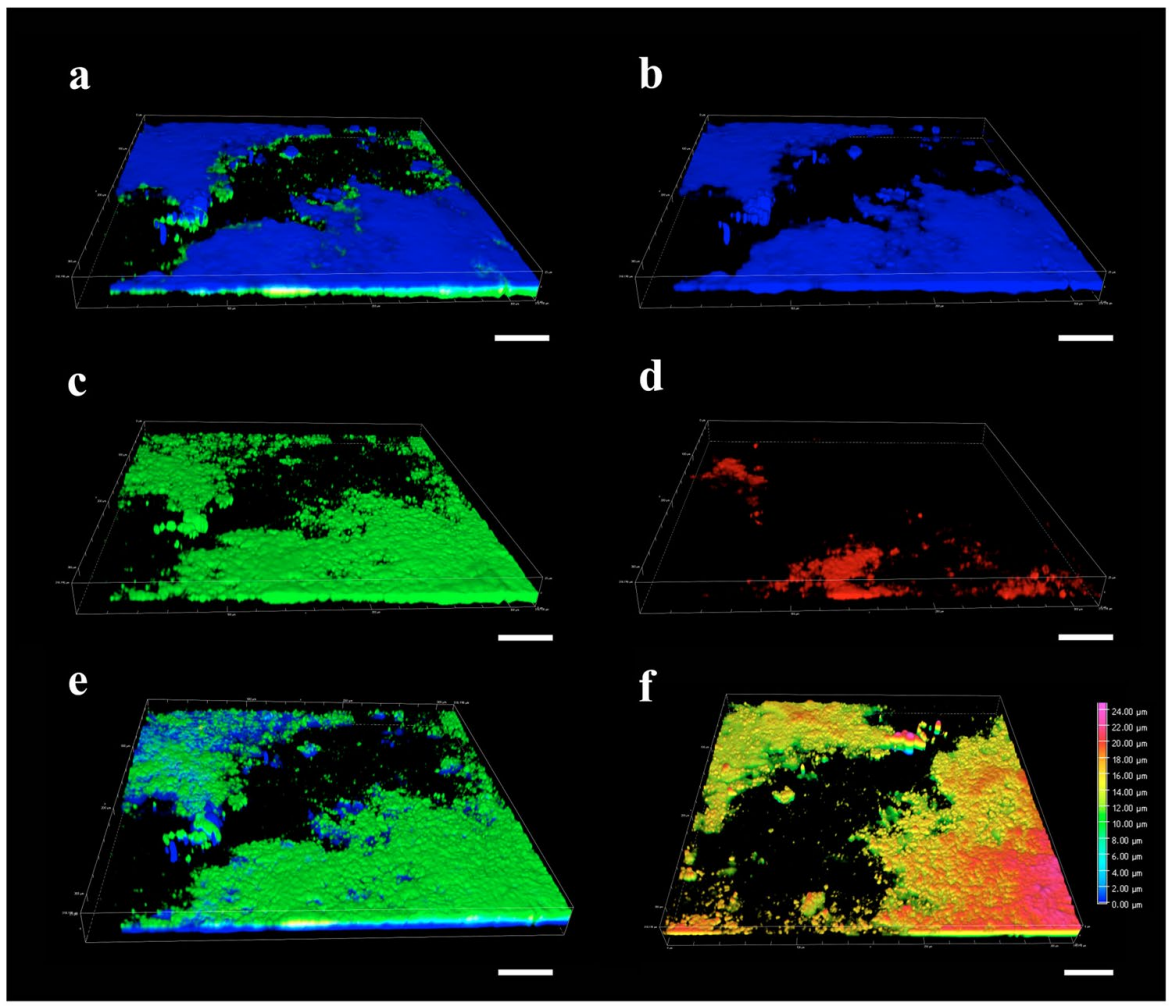
Three-dimensional characterization of *L. crescens* (Lcr) attached biofilms in batch cultures of bBM7 + 0.75 mβc by confocal laser scanning microscope (CLSM). (**a**) Merged image of top view. (**b**) Polysaccharides stained in blue (DAPI filter). (**c**) Live cells stained in green (FITC filter). (**d**) Dead cells stained in red (TRITC filter). (**e**) Merged image of bottom view. (**f**) Depth heat map. Scale bar: 50 µm.

The three dimensional (3D) characterization of seven-day-old Lcr biofilms in bBM7 + 0.75 mβc by confocal laser scanning microscope (CLSM) revealed a layered structure (Fig. 7a) mostly formed by cells attached to the surface of glass cover slips (Fig. 6d), which was better observed from the bottom of the cover slip (Fig. 7e). An extracellular polysaccharide matrix (stained blue by calcofluor white) layer covering and protruding from the cell aggregates was evident (Fig. 7a,b). Small clusters of live cells apparently devoid of extracellular polysaccharide matrix were also occasionally observed (Fig. 7a). Mostly viable Lcr cells were observed in biofilms (Fig. 7c), while dead cells were mostly found in clusters localized in the cell-extracellular polysaccharide matrix interface (Fig. 7d). Lcr biofilms formed with a maximum depth of 24 µm (Fig. 7f).

### Formation of Lcr biofilm under flow conditions in MC

**Figure 8:**
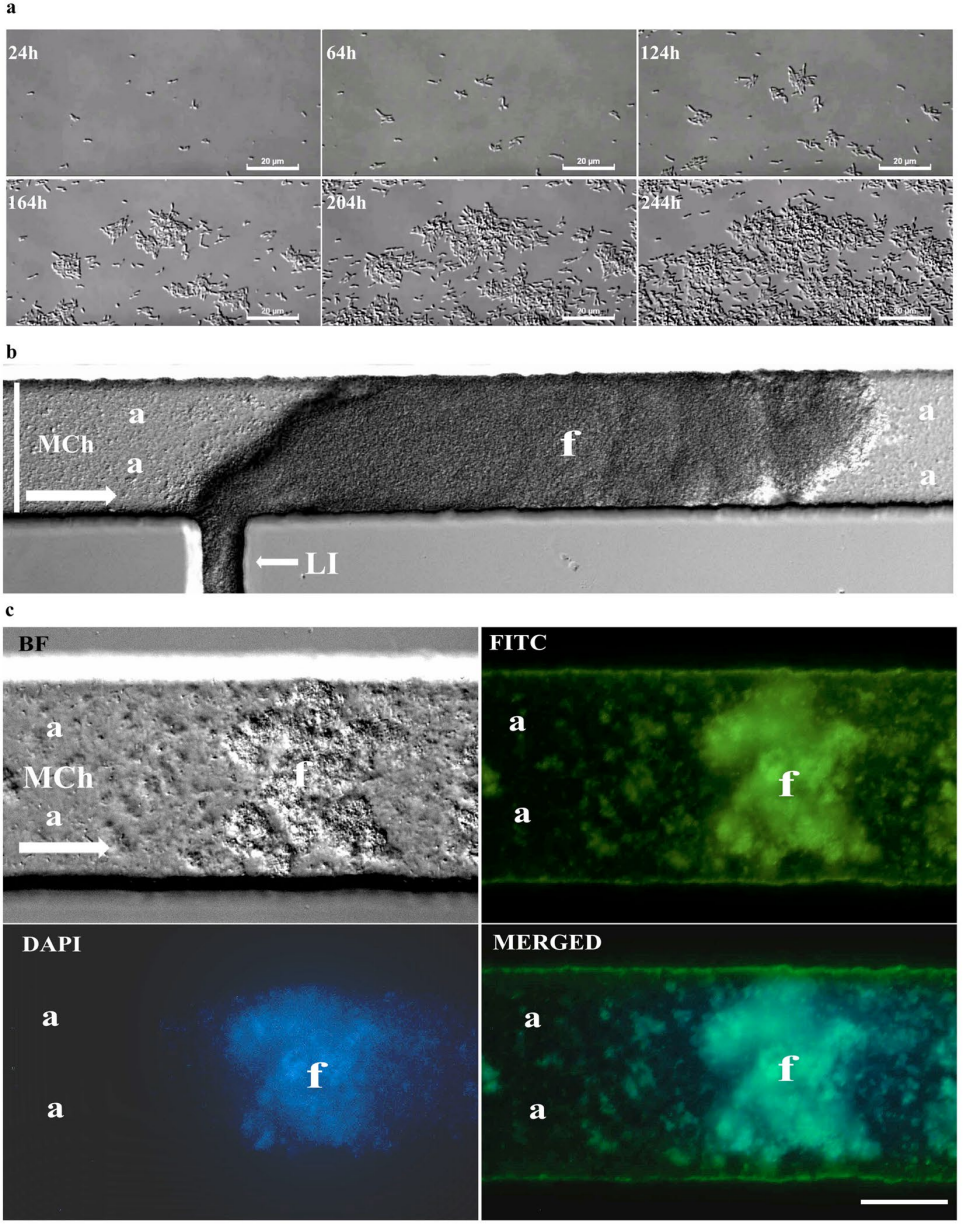
Microscopic characterization of *L. crescens* (Lcr) biofilms in microfluidic chambers (MC) using medium bBM7 + 0.75 mβc. (**a**) Lcr microcolony development over time under flow conditions in the main channel. Scale bar: 20 µm. See also Supplemental Movie 1. (**b**) Different biofilm structures formed by Lcr in MC. (**a**): Areas colonized with Lcr strongly attached biofilms in the main channel (MCh) where a direct constant flow was applied. The arrow in the main channel represents the flow direction. (**f**): floating cell aggregate grown in the lateral inlet (LI) where no direct flow was applied, entering into the main channel (Mch) at 15 dpi. Scale bar: 80 µm. (**c**) Presence of polysaccharides (assessed by calcofluor white staining) in a GFP-Lcr floating cell aggregate originated in the lateral inlet. BF: bright field. FITC: GFP-Lcr cells stained in green. DAPI: Polysaccharides stained in blue. MERGED: Merged images. Scale bar: 40 µm.

In MC assays, where cells are grown under constant flow conditions, Lcr cells were observed mostly in pairs after initial cell attachment that occurred on both glass and polydimethylsiloxane (PDMS) surfaces. After 24–48 h, in the main channel under constant flow conditions, microcolonies started growing in several directions by an initial cell elongation followed by an incomplete cell division, keeping cells closely attached. After initial surface expansion, cells divided more actively from the center of the microcolony, observed by more light refraction from cell aggregates in the center of the microcolony, while a slower cell division activity was observed in cells growing at the edges (Fig. 8a, Movie S1). After ~10 days of culture under flow conditions, large floating cell aggregates were observed arising from the lateral inlets (Fig. 8b). These floating cell aggregates were never observed as originating in the main channel, but only on the side-channels or lateral inlets, where media flow was not actively imposed, but where some limited media movement occurred by disturbances caused by MC handling (Movie S2). The staining of Lcr biofilms inside MC also revealed the presence of polysaccharides associated with the floating cell aggregates that arose from the lateral inlets (Fig. 8c).

## Discussion

‘*Ca*. Liberibacter spp.’ biofilm formation *in vivo* is a complex process that implies adhesion to the luminal face of the psyllid’s gut^23^, intracellular proliferation^14^, and exocytosis through the basal lamina in the form of biofilms, where they putatively produce an extracellular matrix from which cells are spread to cause systemic infection^14,24^. This cycle resembles the development of intracellular bacterial communities described for human pathogens such as uropathogenic *Escherichia coli*^25–27^, *Klebsiella pneumoniae*^27,28^ and *Pseudomonas aeruginosa*^27,29^. In order to form biofilms, ‘*Ca*. Liberibater spp.’ should have the ability to attach to surfaces, proliferate into aggregates and produce a matrix of extracellular polymeric substances. In this work, by defining a model system with optimized cultural conditions for biofilm formation we demonstrated that Lcr: (i) attaches to solid surfaces, (ii) forms cohesive cell aggregates, and (iii) these cell aggregates are embedded in a polysaccharide extracellular matrix resembling exopolysaccharide (EPS). We also evaluated the biofilm formation process in both batch cultures and MC under flow conditions, bringing a valuable approach for future studies including a system (MC) that resembles some of the natural environments where these pathogens inhabit. According to our results, each of these abilities was dependent on media formulation and was influenced by flow conditions.

Cell-surface attachment is the first and a critical step in biofilm formation^30–32^, and is essential for insect colonization and subsequent transmission in insect-transmitted plant pathogens and insect endosymbionts^33–35^. In this study we demonstrated that the presence of the globular protein BSA in culture media blocked Lcr cell-surface attachment and therefore suppressed biofilm formation. BSA is a well-known blocking agent used in enzyme-based immunoassays to avoid nonspecific interactions between biomolecules and solid surfaces^36,37^. BSA is proven to reduce biofilm formation by *Pseudomonas aeruginosa* on plastic surfaces and intravenous catheters^38^ and also blocks surface adhesion of *Staphylococcus aureus* and *S. intermedius* to gold electrodes^39^. Since BSA is one of the major components of FBS, and proved in this study to have antiadhesive properties in MC assays and batch cultures at the same concentration found in bBM7 + FBS medium, we conclude that BSA is the main ingredient interfering with Lcr cell-surface attachment in BM7 medium. Little is known about the antiadhesive properties of compounds present in the plant phloem. However, the vector insect hemolymph contains proteins such as apolipophorins and transferrins, that have been shown to directly affect bacterial-surface adhesion^40–42^ or adsorb to bacterial surfaces^43^. The role of these proteins during ‘*Ca*. Liberibacter spp.’ interactions with insect vectors is unknown, but their antiadhesive properties may influence the transition from the sessile stage to the planktonic phase within the hemocoel, facilitating spread and systemic insect colonization.

After media formulation modifications, Lcr avidly adhered to several materials that included polystyrene, polycarbonate, glass and PDMS. In adhesion force assays in MC, Lcr recorded higher adhesion force values than previously tested species of *X. fastidiosa*^44^ and *Acidovorax citrulli*^45^. The highest adhesion force previously reported using this method was recorded for *Xylella fastidiosa pilB* mutants (strains with only type I pili and no type IV pili) at 204 ± 22 pN^44^, which is still lower than the lowest *Lcr* adhesion force value (of 287.45 ± 25 pN for bBM7 + 0.75 mβc) registered in our assays. Adhesion force comparison also showed that the addition of mβc significantly reduced Lcr adhesion force to MC, although to a much lesser extent than BSA. Lcr genome contains tight-adherence (*tad*/*flp*) genes with orthologues in other ‘*Ca*. Liberibacter spp.’ (Table S1). These proteinaceous structures are highly adhesive and play a major role in cell-surface attachment and cell to cell aggregation for several bacterial species^30^.

After initial cell surface attachment biofilms develop by binary fission of attached cells^21^, therefore bacterial growth rate plays a role in the biofilm formation process. Cultures started from bBM7 + FBS showed a higher total, planktonic and biofilm growth for all treatments tested in comparison to cultures started from bBM7 + 1.0 mβc. FBS contained in bBM7 + FBS culture medium supplies several essential nutrients, trace elements, as well as stabilizing and detoxifying factors needed for maintaining a favorable growth environment^46^. The replacement of FBS with mβc does not seem to fulfill all the nutritional requirements for Lcr growth. Consequently, Lcr grows nutritionally stressed when pre-cultured in the absence of FBS (viz. bBM7 + 1.0 mβc), and therefore, it has a longer lag phase and a lower growth rate when transferred to a medium with optimum conditions such as bBM7 + FBS.

The accumulation of metabolism waste products such as ROS in culture is also a cause of cell stress^47^ and therefore it negatively impacts cell proliferation. Comparison of the Lcr intracellular ROS generation in absence of mβc in bBM7 and at the optimum mβc concentration in bBM7 + 0.75 mβc, showed that mβc significantly reduced Lcr intracellular oxidative stress over time in culture. β-cyclodextrins are cyclic compounds with a hydrophilic surface and a hydrophobic cavity that display remarkable complexation and loading capacity of different guest molecules^48^. These properties have found practical uses as bacterial culture detoxifier compounds and as chemically-defined replacements for serum products necessary for growing fastidious microorganisms^49,50^. Cyclodextrins have been specifically associated with ROS detoxifying effects in *Pseudomonas putida*^51^ and showed a reduction in the expression of oxidative stress-related genes in *Coxiella burnetii*^52^. Our results agree with these previous reports and indicate that mβc addition improved cell proliferation by reducing oxidative stress in culture. At the same time, mβc addition does not cause as drastic reduction in cell-surface adhesion or cell-cell aggregation as FBS does, therefore it brings a biofilm-permissive medium while it provides a more adequate culture formulation for routinely work than bBM7.

The ability of bacterial cells to remain aggregated after cell division is also a main factor during the biofilm formation process^53,54^. Here we demonstrated that Lcr displays a higher cell aggregation in the culture media optimized for biofilm formation, compared with the regular BM7 where it grows planktonically. However, Lcr cells require several days to form aggregates and sediments, while other plant-associated bacteria such as *Dikeya zeae* and *Azospirillum brasilense* can sediment in hours or even minutes^55,56^. A better comparison can be established with *X. fastidiosa*, a vascular fastidious plant pathogenic bacterium with similar temporal growth rate, that aggregates in just hours^57,58^. The examination of *X. fastidiosa* cell aggregation in MC assays reveals that this process is potentiated by an active directional twitching motility of single cells toward main cells aggregates^59^. On the other hand, our assays in MC show that Lcr aggregates passively by cells adhering to each other after binary fission. These differences in motility may account for the divergence in time required for cell aggregation for these two models, despite displaying similar growth rates. Observation of Lcr biofilms by CLSM confirmed that biofilms are mostly formed at the bottom of the flasks. This characteristic is typical of non-motile or motility-defective bacteria^60^, and has been described in bacteria such as *S. aureus*^61^ and *Clostridium perfringens*^62^. Lcr has been initially described as a non-motile bacterium^17^. Lcr genome codifies for components of the type IV pili involved in twitching motility (Table S1), but no signs of active movement were observed in our experimental conditions, therefore, our results in biofilm localization are also consistent with the phenotype observed here.

In biofilms, cell aggregates are structurally supported by a matrix of extracellular polymeric substances mainly composed of polysaccharides^21,63^, thus we also aimed to demonstrate its presence associated with Lcr cell aggregates. In a previous study based in transmission electron microscopy observations, Cicero *et al*.^24^ described the presence of an electron-transparent matrix associated with CLso cell aggregates inside the insect vector *Bactericera cockerelli*, and discussed the potential of both CLas and CLso to produce extracellular polysaccharides. However, nor the bacterial origin or the molecular composition of this matrix were described. In our study, the staining of Lcr cell aggregates with calcofluor white showed that Lcr cells are indeed covered by a polysaccharide matrix. The bacterial origin of this matrix is supported by working with Lcr in pure culture, while its composition is suggested to be based on β-glucans as evidenced by calcofluor white staining^64,65^. Several glycosyl transferases that are involved in oligosaccharides, polysaccharides, and glycoconjugates synthesis, are predicted from Lcr, CLso and CLas genomes^66^. Specially conserved are those involved in capsular polysaccharides, O-antigen lipopolysaccharide (LPS) biosynthesis and ABC-type LPS/glucan transporters that are involved in polysaccharides export (Table S2). These conserved proteins in *Liberibacter* spp. are the core components of the ABC-dependent pathway involved in O-antigen LPS and capsular polysaccharides biosynthesis^67,68^, and are consistent with Lcr biofilm phenotypes observed in our work, containing a low amount of polysaccharides closely attached to the cell aggregates.

Capsular polysaccharides and cell-surface LPS have variable roles in cell adhesion and biofilm formation. Capsular polysaccharides decrease bacterial adhesion to biotic surfaces and have shown to be necessary for biofilm maturation of several human pathogens^69^. Similarly, the O-antigen LPS can improve cell-surface attachment in *Vibrio* spp^70^ and *E. coli*^71^, while its presence decreases motility and biofilm formation in *Bradyrhizobium japonicum*^72^. Nevertheless, both polysaccharide categories have been described to confer resistance to environmental stress factors^73,74^, antimicrobial compounds^75,76^, and remarkably, these polysaccharides are vital for immune system evasion and colonization of intracellular environments by bacterial pathogens^67,77–80^. For nodule-forming rhizobia, mutants in capsule formation were sensitive to higher number of phages, while deficiencies in LPS production reduced the bacteria capacity to overcome plant defense responses during the endosymbiosis process^81^. The ability of *Liberibacter* spp. to produce these polysaccharides may play a role in their symbiosis with their vector insects.

Interestingly, in our study Lcr formed more biofilm under low viability and high oxidative stress conditions. Additionally, surface-attached biofilms were observed only under constant flow condition, while loose aggregates were formed only in the lateral inlets where flow was not directly imposed. Some of these findings may suggest explanations for the role of ‘*Ca*. Liberibacter spp.’ cell-surface attachment in the gut lumen and aggregation and polysaccharide formation after exocytosis through the basal lamina^24,82^. In the niche of the luminal face of the insect gut, ‘*Ca*. Liberibacter spp.’ cells are constantly exposed to continuous flow therefore a strong cell-surface attachment is priority over multiplication or extracellular matrix formation to ensure retention in the host and subsequent endocytosis. On the other side, inside the epithelial cells or the insect hemolymph, nutrients are abundant^83,84^ and ‘*Ca*. Liberibacter spp.’ undergo extensive proliferation^24^, however, these pathogens have to face the insect immune system^85^, and an increased ROS production resulting from the programmed cell death induced in the midgut cells^82^. Oxidative stress and biofilm formation are well documented co-regulated processes in bacteria^86–88^, thus the ability to aggregate and produce extracellular polymeric substances may have a protective role against insect natural defenses as for other intracellular bacterial pathogens. The reduction in shear stress in the wider hemocoel cavity, compared with the luminal gut face, may also allow the formation of larger cell aggregates, like those formed under lower flow rates in MC for Lcr. In conjunction, the abilities of strong cell attachment, cell aggregation and exopolymeric matrix formation described in this work for Lcr in continuous flow and batch conditions, may represent adaptive advantages for ‘*Ca*. Liberibacter spp.’ in their insect host for survival, proliferation and transmission.

## Methods

### Bacterial strain and culture conditions

*L. crescens* strain BT-1^16^ was grown on BM7 (also referred here as bBM7 + FBS, see above) solidified with 15% agar^16^ at 28 °C. FBS was obtained from HyClone Laboratories (South Logan, UT, USA). Cultures were started from cryopreserved stocks (bBM7 + FBS with 20% glycerol, −80 °C) by first plating onto bBM7 + FBS agar plates and incubating at 28 °C for 6–7 days. Next cultures were re-streaked for a second passage in the same media for additional 6 days, prior to starting each experiment.

### Media formulations

Media formulations used in this work are summarized in Table 1. A basal medium, bBM7, consisting of BM7 minus FBS was used for Lcr bacterial suspension preparation and designated as a base for subsequent media modifications. Initially, solid agar (15%) plates of bBM7 medium containing 1 g/l of mβc (SIGMA-ALDRICH, St. Louis, MO, USA) (bBM7 + 1.0 mβc) were used for optimization experiments (Figs 1b,d and 2b,d and 3b). Methyl-β-cyclodextrin initial concentration of 1 g/l was selected based on previous studies that demonstrated the effective replacement of FBS by the less costly mβc when culturing other intracellular fastidious bacteria^52^. After culture media optimization (see below), medium referred here as bBM7 + 0.75 mβc was prepared by supplementing bBM7 with 0.75 g/l of mβc and used for biofilm characterization. Since BSA is a component of FBS and known to have bacterial antiadhesive properties^38^, cell-surface attachment was assessed using bBM7 compared with bBM7 supplemented with the average concentration of BSA contained in bBM7 + FBS (a.k.a BM7) of 3.5 g/l. This last formulation was denoted as bBM7 + BSA.

### Media optimization for biofilm formation

Optimal mβc and FBS concentration values for biofilm growth were determined in polystyrene 96 well plates (COSTAR^®^, Kennebunk, ME, USA). To explore the influence of the initial growth media on subsequent bacterial growth, assays were started from either bBM7 + FBS (a.k.a BM7) or bBM7 + 1.0 mβc agar plates. Wells along the outer edges of the 96 well plates were initially filled with 250 µl of sterile deionized water to mitigate desiccation effects. Ten μL of the Lcr bacterial suspension in bBM7 (OD_600 nm_ = 1) was added to 190 µl of bBM7 supplemented with mβc in a concentration gradient ranging from 0 to 1 g/l in 0.25 g/l increments. Ten μL of the Lcr bacterial suspension in bBM7 (OD_600 nm_ = 1) was added to 190 µl of bBM7 supplemented with increasing FBS amounts to reach final concentrations of 3, 6, 9, 12 and 15% for each media formulation.

Six blank wells and six Lcr cultures replicates were included for each treatment. The experiments were repeated twice independently. Plates were wrapped with parafilm and incubated at 28 °C and 150 rpm for 6 days. Growth kinetics for each treatment were determined by daily turbidity measurements at 600 nm (OD_600_), using a Cytation 3 Image Reader spectrophotometer (BioTek Instruments Inc., Winooski, VT, USA).

In a second set of experiments, late exponential phase cultures (6 days-post-inoculation, dpi) under the same treatments described above were used for total, planktonic and biofilm growth quantification. “Total” growth was determined by turbidity measurements as described above. “Planktonic” and “biofilm” growth were quantified using a crystal violet assay^89^. Briefly, 150 µl of liquid media containing suspended cells were transferred to a new 96 well plate for OD_600_ measurement, and this value was considered “planktonic” growth. The remaining liquid media in the initial 96-well plate culture was poured and to remove remaining planktonic cells, the wells were gently rinsed with 230 µl of molecular-grade water 3 times, using a multichannel pipette. Then, 230 µl of a 0.1% crystal violet solution was added to each well and the plate was kept at room temperature for 20 minutes. After this time, the crystal violet was removed with a multichannel pipette and the wells were gently washed with molecular-grade water as described above to remove the remaining crystal violet. After washing, 230 µl of 95% ethanol were added to each well and incubated under agitation (150 rpm) for 5 min. OD_600_ of each well was read as described above, and it was considered as “biofilm” growth.

Significant differences in total, planktonic, and biofilm growth among treatments were determined by standard ANOVA and mean comparisons among treatments were performed using Fisher’s protected least significant difference (LSD) test at *P* < 0.05, using SIGMA Plot Software, Version 11.0 (Systat Software, Inc., San Jose CA, USA).

### Cell viability measurements

Lcr cell viability was measured with the alamarBlue^®^ cell proliferation and viability reagent (Bio-Rad Laboratories Inc, Hercules, CA, USA). Briefly, Lcr cultures were inoculated in an mβc concentration gradient as previously described and incubated for six days at 28 °C, 150 rpm. Then, 20 µl of alamarBlue^®^ was added to Lcr cultures and allowed to incubate for four additional hours under the same growth conditions. To avoid cell interference with the absorbance readings, each plate was centrifuged at 4000 rpm for 10 min and 150 µl of the cell free supernatant was transferred to a fresh plate. Absorbance measurements were performed at 600 and 570 nm wavelengths using a Cytation 3 Image Reader spectrophotometer. Lcr viability at each mβc concentration was assessed as the Percentage Reduction of alamarBlue (PRAB), following the absorbance-based method described by the manufacturer. Briefly, the equation $[PRAB=(O2\times A1)-(O1\times A2)/(R1\times N2)-(R2\times N1)\ast 100]$ was used, where O1 = molar extinction coefficient (E) of oxidized alamarBlue^®^ at 570 nm; O2 = E of oxidized alamarBlue^®^ at 600 nm; R1 = E of reduced alamarBlue^®^ at 570 nm; R2 = E of reduced alamarBlue^®^ at 600 nm; A1 = absorbance of test wells at 570 nm; A2 = absorbance of test wells at 600 nm; N1 = absorbance of negative control well (media plus alamarBlue^®^ but no cells) at 570 nm; and N2 = absorbance of negative growth control well (media plus alamarBlue^®^ but no cells) at 600 nm. Four replicates were considered per treatment, and experiments were repeated twice independently.

To identify the variation in biofilm formation and planktonic growth for each culture condition, and determine its relationship with cell viability, biofilm absorbance values assessed by the crystal violet assay (see above) were divided by the planktonic absorbance values for each replicate and denoted as biofilm/planktonic ratios for each mβc treatment. Significant differences among cell viability (quantified as PRAB), as well as between biofilm/planktonic ratios for each treatment, were determined by standard ANOVA, and mean comparisons among treatments were performed using Fisher’s protected LSD test at *P* < 0.05 using SIGMA Plot Software. The medium formulation with the mβc concentration value that displayed the optimum combination of growth rate, biofilm formation and cell viability (bBM7 + 0.75 mβc, Table 1) was used for subsequent experiments.

### Intracellular reactive oxygen species quantification

The general oxidative stress indicator dye CM-H_2_DCFDA (Life Technologies Corporation, Eugene, OR, USA) was used to quantify the generation of intracellular ROS by Lcr in different media. Briefly, six-day-old Lcr cultures in bBM7 + FBS agar plates were washed and suspended in pre-warmed (28 °C) phosphate-buffered saline (PBS, pH 7.4) at a final OD_600_ = 1. CM-H_2_DCFDA was added to the bacterial suspension at 10 μM and incubated for 20 min at 28 °C, 150 rpm in the dark to allow dye penetration. Then the dye was removed from the suspension by centrifugation at 8,000 rpm for 5 min and the pellet was washed twice with pre-warmed PBS. Then, bacterial pellets were resuspended in different media and incubated under the same conditions described above for 4 hrs. The fluorescent signal intensity for each treatment was measured right after pellet suspension and every hour for 4 h using a Cytation 3 Image Reader spectrophotometer (BioTek Instruments Inc.) at an excitation and emission wavelength of 492 nm and 517 nm, respectively. Lcr intracellular ROS production on each media formulation was determined as the average fluorescence intensity for three replicates per treatment. Significant difference in fluorescence emission between the treatments at each time point was calculated by standard ANOVA and mean comparisons among treatments were performed using Fisher’s protected LSD test at *P* < 0.05 using SIGMA Plot Software. Experiments were repeated 2 times.

### Strength of bacterial surface attachment in microfluidic chambers

Lcr cell attachment force for different media was assessed in MC as previously described (Cruz *et al*.^90^; De La Fuente *et al*.^44^) with some modifications. Briefly, MCs were built using photolithography, by using PDMS and glass for the main surfaces, and tigon tubing connected to syringes for delivery of media and bacterial suspensions, as previously described^44^. MCs were initially filled with each media formulation through the main channels and air bubbles were removed by pumping the media through the main channels at a flow rate of 40 µl/min using an automated syringe pump (Pico Plus; Harvard Apparatus, Holliston, MA, USA). Then, culture media flow was stopped, and Lcr bacterial suspensions on the respective tested media formulation (OD_600_ = 1) were aseptically loaded into sterile 1 ml syringes, and the syringes were connected to the lateral inlets avoiding bubble generation. Then, both culture medium and bacterial cultures were inoculated at a flow speed of 0.25 µl/min for 1 h. Subsequent steps were performed as previously described by Cruz *et al*.^90^. Significant differences between cell attachments registered for each media formulation was determined by Student’s *t*-test at *P* < 0.05, using SIGMA Plot Software.

### Bacterial cell-to-cell aggregation assessment

The aggregation of Lcr cells was assessed as previously described^57^ in the selected culture media formulation for biofilm formation (bBM7 + 0.75 mβc) and bBM7 + FBS. In summary, Lcr cells grown on bBM7 + FBS agar plates for five days were harvested in PBS buffer to an OD_600_ = 1.0, and five ml aliquots were inoculated in both 45 ml of fresh bBM7 + FBS and bBM7 + 0.75 mβc broth in Erlenmeyer flasks to assess Lcr cell aggregation in each media. Lcr cultures were grown for seven days at 28 °C and 150 rpm, when cells (including any biofilm formation) were centrifuged at 4000 rpm for five minutes (room temperature) and resuspended to an OD_600_ = 1.0 in a final volume of 10 ml in conical tubes, using the same media broth where they were already grown (considered time 0). Every 24 h for seven days (total of 168 hours), without disturbing cell suspensions, 1 ml of the surface of the culture of each tube was taken to measure OD_600_ of samples. Fresh bBM7 + FBS and bBM7 + 0.75 mβc media broth were used as blanks for the assay. The level of cell aggregation is inversely proportional to the OD_600_ of the supernatant, due to cell sedimentation at the bottom of the tubes. The settling rate of cells in each growth condition was calculated using the formula {settling rate = [ln (OD_600_ 0 hpi) – ln (OD_600_ 168 hpi)/time; hpi – hours-post-inoculation}, as described previously^58^. The assay was performed as four independent biological replicates. Significant differences between cell aggregations in the different growth conditions were calculated by Student’s *t*-test at the significance level of *P* < 0.05, using SIGMA Plot Software.

### Green Florescent Protein (GFP)-marked L. crescens

*L. crescens* strain BT-1 was marked with green fluorescent protein (*gfp*) and a kanamycin (Kan)-resistance genes via marker-interruption of a nonessential target locus, *lcrRIP* (Type I restriction endonuclease subunit R; B488_RS03405) in the chromosome, following the strategy outlined in Castañeda *et al*.^91^. The *P*_*trp*_::*gfp* cassette (946 bp) from pUFZ75^92^ was amplified using primers 5′ TAAGATCTGAACGCGTATGAGCTCTAAGAAGCTTGGCAAATAT 3′ and 5′ *TCATACGCGTTCAGATCTTA*GCTCGAATTCCTATTTGTATAGT 3′. A partial (985 bp) DNA fragment internal to the coding region of *lcrRIP* was amplified using primers 5′ *TAAGATCTGAACGCGTATG*ACACCACGCGCAGGAT ATTA 3′ and 5′ GGATCCTGAGCCAGTACAAAGC GTATCCTGCGTA 3′. The two amplicons were fused together using splice-overlap PCR (overlapping primer sequences marked in *italics*) and cloned into pCR^®^2.1-TOPO (Kan^R^, Invitrogen, Carlsbad, CA, USA). The resulting plasmid pMJ029 was used as a suicide plasmid and transformed into Lcr strain BT-1 exactly as described by Jain *et al*.^20^. The GFP marked and Kan-resistant transformed Lcr strain (BT-1/pMJ029 GFP, Kan^R^; referred here as GFP-Lcr) was grown on bBM7 + FBS plates with 4.5 mg/l Kan at 28 °C. The strain GFP-Lcr was stored at −80 °C and cultured on selective media for 7–8 days and then re-streaked for another six days prior to conducting each experiment.

### Microscopic characterization of Lcr cell-cell aggregates and surface-attached biofilms

Microscopic Lcr biofilm characterization was performed in 24 well plates (VWR International, LLC, PA, USA). Lcr formed biofilms at the bottom of the culture flasks, as described for other non-motile facultative anaerobes^61,62,93^. In order to collect biofilms from the bottom of the cultures, 0.8 × 0.8 cm glass pieces were cut from borosilicate cover glass slides (Fisherbrand^®^, Pittsburng, PA, USA), autoclave-sterilized for 15 min and dried for 30 min at 80 °C. After cooling, the glass pieces were aseptically placed at the bottom of sterile polystyrene 24 well plates. Then, two ml of Lcr cultures in bBM7 + FBS and bBM7 + 0.75 mβc (OD_600_ = 0.5), were inoculated by quadruplicate and incubated at 28 °C and 150 rpm for eight days. After incubation, one ml of the liquid was carefully removed from each culture without disturbing the settled cell aggregates. All cultures were observed under a Zeiss Stemi 508 stereo microscope (Göttingen, Germany) at 10X magnification. Images were captured with a Carl Zeiss GmbH microscopy camera (Göttingen, Germany) controlled by the ZEN lite software (Carl Zeiss Microscopy GmbH, Jena, Germany).

Cell clumps that formed at the bottom of the cultures of the GFP-Lcr strain were carefully transferred altogether with one ml of culture medium to a well in a fresh plate using 1 ml wide bore pipette tip. Then, 10 µl of calcofluor white (St. Louis, MO. USA) and 10 µl of KOH 10% were added and incubated for five minutes in the dark, following manufacturer’s guidelines. After staining, the cell clumps were recovered with sterile tweezers without disrupting them and sandwiched between two borosilicate coverslips. Image analysis was performed using a Nikon Eclipse A1 CLSM (Nikon, Melville, NY, USA) using a 60X oil immersion objective. To detect cells and extracellular polysaccharide matrix, excitation wavelengths of 528 and 590 nm were used, respectively.

To characterize attached biofilms, six µl of an equal volume mix of both reagents of the LIVE/DEAD^®^ BactLight Bacterial Viability Kit (Invitrogen, Eugene, OR. USA) were added to each glass slide-containing well with the wild-type Lcr BT-1 strain. The 24 well plates were then wrapped in aluminum foil and incubated at 150 rpm and 28 °C for 15 min. After incubation, 10 µl of calcofluor white and 10 µl of KOH 10% were added to each well and incubated for additional 5 minutes under the same conditions. Each slide was then aseptically removed from the well, gently washed three times with sterile deionized water and placed on top of a borosilicate slide with the biofilm facing up. A drop of water was added over the attached biofilms to maintain the 3D structure and to avoid desiccation. Image analyses were performed using a Nikon Eclipse A1 CLSM (Nikon) using a 40X distance objective. All three dyes used, namely propidium iodide, SYTO 9 and calcofluor white, were detected at excitation wavelengths of 528, 590, and 370 nm, respectively. To analyze the 3D structure of the biofilm, one µm interval z-series were automatically captured in a deep range of 25 µm. Images were acquired with a CoolSnap HQ2 camera (Photometrics, Tucson, AZ, USA) and processed with NIS-Elements AR software, version 3.0 (Nikon).

### Lcr biofilm formation in microfluidic chambers (MC)

MC design and fabrication was performed as previously described^44^. The channels were initially filled with bBM7 + 0.75 mβc using an automated syringe pump (Pico Plus; Harvard Apparatus, Holliston, MA). Lcr BT-1 wild type strain bacterial suspensions in bBM7 + 0.75 mβc (OD_600_ = 0.5) were injected for one hour to obtain attached cells. After a critical number of attached cells was observed (app. >10 cells/100 µm^2^), bacterial cultures inlets were clamped with surgical scissors, and the flow rate of the media was maintained constant at 0.25 µl/min for two weeks. The MC was mounted onto a Nikon Eclipse Ti inverted microscope (Nikon) and observed with a 40X objective using phase contrast and Nomarski differential interference contrast (DIC) optics. Cell division over time was recorded using time-lapse video imaging microscopy. Image acquisition was performed automatically every 10-min using a Nikon DS-Q1 digital camera (Nikon) controlled by NIS- Elements software version 3.0 (Nikon). In order to detect and colocalize cells and polysaccharides inside MC, a second set of experiments was performed using the GFP-Lcr strain. Inoculation, flow rate parameters and growth assessment were performed as described above. After 10 days of experiment, the syringe with bBM7 + 0.75 mβc culture medium was replaced with one ml of bBM7 + 0.75 mβc supplemented with five µl each of KOH 10% and calcofluor white, and the mix was allowed to flow inside the MC at the same flow rate used for growth for six hours. Image capture was performed using a Nikon Eclipse Ti inverted microscope (Nikon) and observed with a 40X. To detect both the Lcr-GFP cells and the polysaccharides stained by calcofluor white excitation wavelengths of 528 nm (FITC filter) and 370 nm (DAPI filter), respectively, were used. Image analysis was conducted using the NIS- Elements software version 3.0. At least three independent experiments were performed with each strain.

## Supplementary information

Supplementary files

Movie S1

Movie S2
