# Supplementary material for: Liberibacter crescens biofilm formation in vitro: establishment of a model system for pathogenic ‘Candidatus Liberibacter spp.’
Source: Sci Rep. 2019 Mar 26;9:5150. doi: 10.1038/s41598-019-41495-5 (PMC6435755; doi:10.1038/s41598-019-41495-5)
Supplement: Supplementary file 1 — Supplementary files [file 41598_2019_41495_MOESM1_ESM.pdf]

Supplementary Material

***Liberibacter crescens* biofilm formation in vitro: establishment of a model system for pathogenic '*Candidatus* Liberibacter spp.'**

Eber Naranjo, Marcus V. Merfa, Virginia Ferreira, Mukesh Jain, Mike J. Davis, Ofir Bahar, Dean W. Gabriel, and Leonardo De La Fuente*

*Corresponding Author
lzd0005@auburn.edu

1. Supplementary tables

TABLE S1. Accession numbers for the amino acid sequences of proteinaceous surface appendages predicted in *L. crescens* genome and their homologs in other *Liberibacter* spp.

Annotation	Lcr BT 1	CLas psy62	CLso ZC1	CLam S. Paulo
Flp pilus assembly protein RcpC/CpaB	B488_12850	CLIBASIA_03075	CKC_00710	lam_225
Type IV pili component	B488_12830	CLIBASIA_03065	CKC_00700	lam_227
Flp pilus assembly protein, ATPase CpaF	B488_12810	CLIBASIA_03055	CKC_00690	lam_229
Type II/IV secretion system protein TadC	B488_12790	CLIBASIA_03040	CKC_00680	lam_231

Lcr: *Liberibacter crescens*; **CLas:** '*Candidatus* Liberibacter asiaticus'; **CLso**: '*Candidatus* Liberibacter solanacearum'; **CLam**: '*Candidatus* Liberibacter americanus'.

TABLE S2. Accession numbers for the amino acid sequences of proteins involved in polysaccharide biosynthesis and export predicted in *L. crescens* genome and other *Liberibacter* spp.

Annotation/Function	Lcr BT 1	CLas psy62	CLso ZC1	CLam S. Paulo
Glycosyl transferase PRK10714 super family/ Cell envelope biosynthesis	WP_015272446.1	-	-	-
YqgM like Glycosyl transferase/unknown	WP_015272549.1	WP_015452359.1	WP_013461767.1	-
HAD like glycosyl transferase/ Haloacid dehalogenase	WP_015273097.1	-	-	-
GT like family 2-3/ Bacterial capsule biosynthesis	WP_015273098.1	-	-	-
Glycosyl tranferase RfaB superfamily/ Cell wall/membrane/envelope biogenesis	WP_015273270.1	-	-	-

Lcr: *Liberibacter crescens*; **CLas:** '*Candidatus* Liberibacter asiaticus'; **CLso**: '*Candidatus* Liberibacter solanacearum'; **CLam**: '*Candidatus* Liberibacter americanus'. **-**: Not present.

TABLE S2. Accession numbers for the amino acid sequences of proteins involved in polysaccharide biosynthesis and export predicted in *L. crescens* genome and their homologs in other *Liberibacter* spp. (Continuation).

**Annotation/ Function**	**Lcr BT 1**	**CLas psy62**	**CLso ZC1**	**CLam S. Paulo**
RfbC like Glycosyl transferase/ O-antigen biosynthesis	WP_015273419.1	WP_015452749.1	WP_080550997.1	-
Glycosyl transferase/D-glucosamine synthase	-	WP_015452749.1	-	-
Glycosyl transferase family 25/ lipooligosaccharide biosynthesis	-	WP_015452945.1	WP_044054317.1	-
Lbpt family ABC-type LPS transporter/ Export system, ATPase component. Cell wall/membrane/envelope biogenesis	WP_015272549.1	WP_015452420.1	WP_013461564.1	WP_007 556704.1
Predicted permease YjgP/YjgQ family Lipopolysaccharide export	WP_041770545.1	WP_015452349.1	WP_045960892.1	WP_007 556928.1

Lcr: *Liberibacter crescens*; **CLas:** '*Candidatus* Liberibacter asiaticus'; **CLso**: '*Candidatus* Liberibacter solanacearum'; **CLam**: '*Candidatus* Liberibacter americanus'. **-**: Not present.

2. **Caption for supplementary videos**

Movie S1. Time-lapse video showing *Liberibacter crescens* (Lcr) microcolony development over time under flow conditions in microfluidic chambers (MC). The MC was filled with bBM7+0.75mβc liquid media and inoculated with an Lcr suspension in the same culture media. In the video the flow runs from left to right at 0.25 µl/min. Scale bar: 20 µm.

Movie S2. Real-time video showing a *Liberibacter crescens* (Lcr) polysaccharide-cell aggregate in microfluidic chambers (MC). The MC was filled with bBM7+0.75mβc liquid media and inoculated with an Lcr suspension in the same culture media. Scale bar: 50 µm. In the second part of the video polysaccharides are stained in blue with calcofluor white and observed under the fluorescence microscope using an excitation wavelength of 370 nm.
